# From DNA-Encoded Chemistry to Tumor-Targeted Small Molecule Therapeutics

**DOI:** 10.3390/ph19081137

**Published:** 2026-07-23

**Authors:** Samuele Cazzamalli, Dario Neri

**Affiliations:** 1 R&D Department, Philochem AG, CH-8112 Otelfingen, Switzerland; 2 Department of Chemistry and Applied Biosciences, Swiss Federal Institute of Technology, CH-8093 Zurich, Switzerland; 3 Philogen S.p.A., 53100 Siena, Italy

**Keywords:** diagnostic radiotracers, therapeutic radiopharmaceuticals, theranostics, translation into clinics and first-in-human studies, DNA-encoded chemical libraries

## Abstract

Unlike conventional screening methods, which are limited by library size, DNA-encoded chemical library (DEL) technology enables the simultaneous interrogation of billions of compounds, each uniquely barcoded with DNA tags. These libraries can be screened through affinity-based capture and decoded using high-throughput sequencing. DEL-derived ligands can be conjugated to cytotoxic agents or radioactive isotopes, facilitating the creation of highly selective therapeutics that target diseased cells while minimizing off-target effects. This article explores the use of DELs for identifying high-affinity small organic ligands for the development of small molecule–radio conjugates (SMRCs) and small molecule–drug conjugates (SMDCs).

## 1. Introduction

Conventional anticancer chemotherapeutic agents are, in most cases, small organic molecules that preferentially kill (or inhibit the growth of) rapidly dividing cells [1]. While it is true that tumor cells grow more rapidly than many other cells in the body, they are not the only cells in proliferation. For this reason, common side effects of cancer chemotherapy may include myelotoxicity, hair loss, damage to mucosae (to mention just a few), as such side effects correspond to bodily structures in constant rapid regeneration. This selectivity limitation of chemotherapy, which we could consider “biochemical” in nature, is not the only limitation of the therapeutic approach. Indeed, virtually all anti-cancer small organic drugs do not preferentially localize at the tumor site [2,3]. This pharmacokinetic limitation may lead to toxicity to normal organs and prevent the dose escalation to therapeutically relevant regimens. A rational avenue to improve the therapeutic index of potent (but not selective) anticancer drugs (and, for what matters, of therapeutic radionuclides) consists of the coupling of those active payloads to small organic ligands, specific to accessible and selective tumor-associated markers. This article focuses on the applications of DEL technology to the field of ligand-based pharmacodelivery.

The discovery of molecules capable of high-affinity and selective recognition of target proteins of biomedical interest is a central challenge in pharmaceutical sciences. Such a challenge becomes even more relevant (and more difficult!) in the field of ligand-based pharmacodelivery, as ligands with ultra-high binding affinity to the cognate target (i.e., with a *K_d_* dissociation constant value below 1 nM) are needed to obtain an efficient preferential enrichment at the site of disease [4]. This empirical observation can also be justified based on simple theoretical considerations, outlined below. When considering a simple chemical binding equilibrium, in which a ligand **L** binds to a cognate target **T**, the process can be described by the following chemical equation:

**L** + **T** ⇌ **LT**

(L: ligand; T: target; LT: ligand-target comples)

The corresponding dissociation constant *K_d_* is equal to the ratio of the kinetic dissociation and association constants *k_off_* and *k_on_*:

$$K_{d}=\frac{k_{off}}{k_{on}}$$

In turn, *k_off_* is equal to the ratio between ln2 (i.e., 0.693.) and the dissociation half-life of the ligand–target complex. As an upper limit exists for *k_on_* values in physiological conditions, corresponding to the diffusion control limit (in most cases limiting *k_on_* to values of 10^6^ s^−1^M^−1^, or lower), this automatically implies certain ranges for *k_off_* values, in correspondence to certain *K_d_* dissociation constant values. For example, a *K_d_* value of 1 nM and a *K_on_* value of 10^6^ s^−1^M^−1^ implies a *k_off_* value of 10^−3^ s^−1^, which in turn corresponds to a kinetic half-life of the ligand–target complex of approximately 11 min. Even though rebinding procedures can prolong the ligand residence at the site of disease if the target is particularly abundant, or by exploiting multivalent binding modalities, it is clear that ligands with *K_d_* values in the subnanomolar concentration range will be preferable in order to achieve a long residence time at the site of disease.

DEL technology is ideally suited for the discovery of ultra-high-affinity protein ligands, for at least two reasons:

(i) DEL technology allows the construction and screening of compound libraries of unprecedented size and quality. From a theoretical consideration, an increase in library size is expected to convert into an increased probability of discovery of high-affinity ligands [5]
(ii) DEL technology facilitates affinity-maturation strategies, starting from initial hits that need to be expanded.

**Figure 1:**
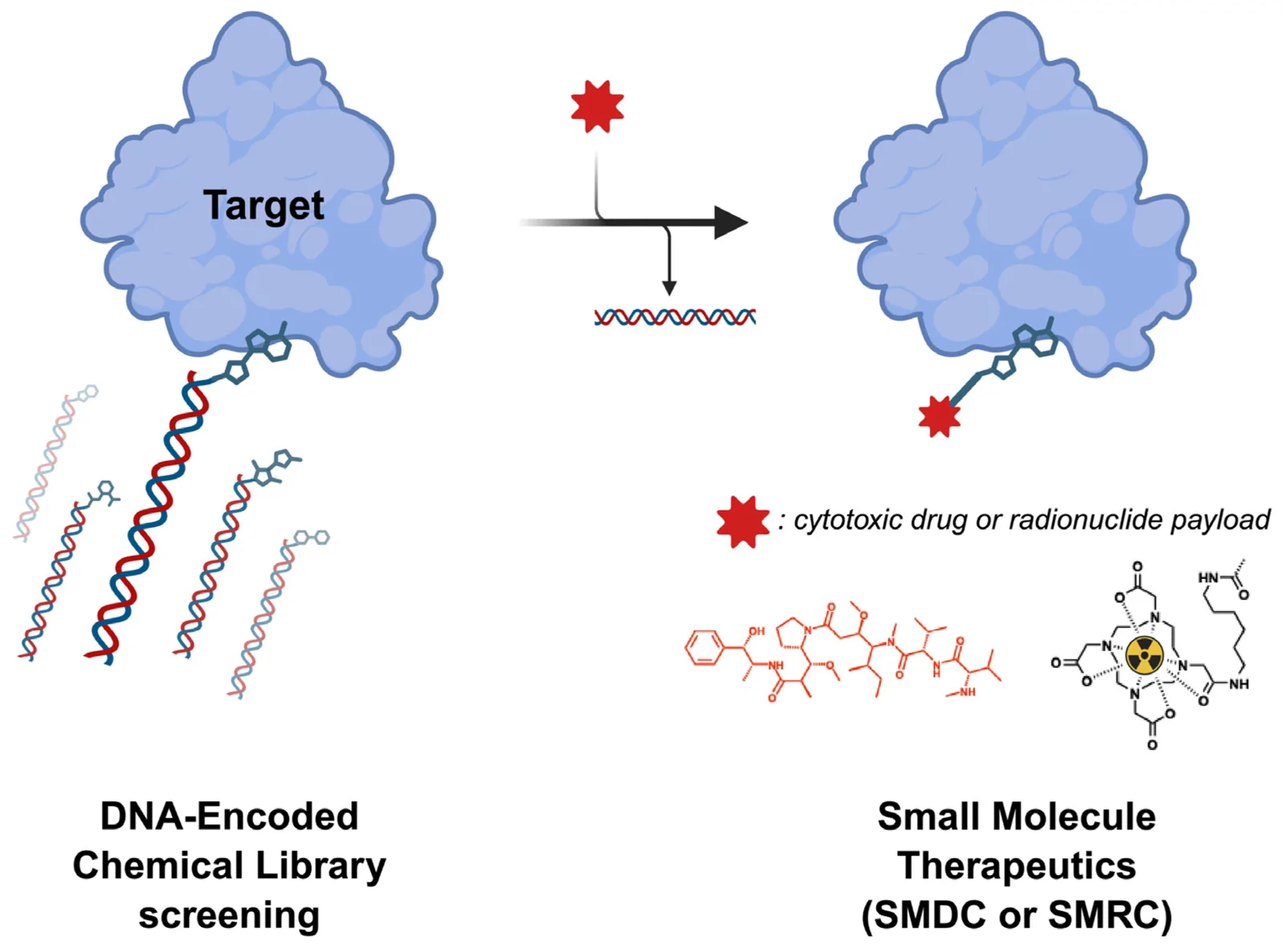
From DNA-encoded chemical libraries to tumor-targeting ligands. DNA-encoded libraries (DELs) comprising diverse small-molecule derivatives can be screened against a protein of interest (“Target”) to identify small molecule ligands interacting with the target with high specificity and affinity. Enriched library members can be resynthesized without the DNA tag and conjugated to a highly cytotoxic drug or a radionuclide payload. These resulting small molecule therapeutics have the potential to selectively accumulate in target-expressing tumor lesions, highlighting a versatile strategy for the discovery of targeted small molecule–drug conjugates (SMDCs) or small molecule–radio conjugates (SMRCs). Created in BioRender. Rotta, G. (2026) https://BioRender.com/ph96sbe (accessed on 24 June 2026).

It is important to mention that the DNA attachment site of small molecules represents a suitable site for replacing the DNA barcodes with suitable payloads, such as drugs or radionuclide complexes, Figure 1.

In the next section, we briefly discuss why small organic ligands may be preferable to antibodies for pharmacodelivery applications.

## 2. Replacing Tumor-Targeting Antibodies with Small Organic Ligands

Monoclonal antibodies (mAbs) have long been the cornerstone of targeted cancer therapy, offering high specificity for tumor-associated antigens (i.e., target proteins expressed selectively in tumor lesions) and enabling the selective delivery of cytotoxic payloads or radionuclides to malignant tissues [6,7,8]. However, the limitations of antibody-based approaches have spurred increasing interest in small organic ligands as alternative targeting vectors [4,9]. These low-molecular-weight molecules offer several intrinsic advantages over antibodies, making them particularly attractive for next-generation therapeutic conjugates such as small molecule–drug conjugates (SMDCs) [4,10,11] and small molecule–radio conjugates (SMRCs) [7].

While monoclonal antibodies have the distinct advantage of being readily generated against virtually any target of interest with high affinity and specificity through established immunization or selection techniques (e.g., phage display technology) [12], small molecule discovery has traditionally faced greater challenges in achieving comparable target coverage. The advent of DNA-encoded libraries (DELs) has significantly expanded the chemical search space and discovery throughput, enabling the screening of billions of compounds against a given protein target [13,14,15,16]. However, despite their transformative potential, DELs still face technical and conceptual limitations that must be addressed before they can consistently yield high-affinity, selective ligands for a broad range of targets, particularly those considered “undruggable” or lacking well-defined binding pockets [17]. Improvements in DEL design, such as the incorporation of more structurally diverse scaffolds, improved encoding chemistries, and refined selection protocols, are actively being pursued to enhance the productivity of the technology [18,19,20,21]. For example, Li and colleagues recently explored a short-splint RNA-mediated single-stranded DNA (*ss*DNA) ligation system to address challenges in constructing and selecting single-stranded DELs (*ss*DELs) on living cells, a technology that enabled them to isolate a novel picomolar ligand against Carbonic Anhydrase XII [21]. The ongoing evolution of DEL technology holds promise for eventually bridging the gap with antibody technologies and enabling the discovery of potent, selective small ligands across a much broader portion of the proteome [22,23].

**Figure 2:**
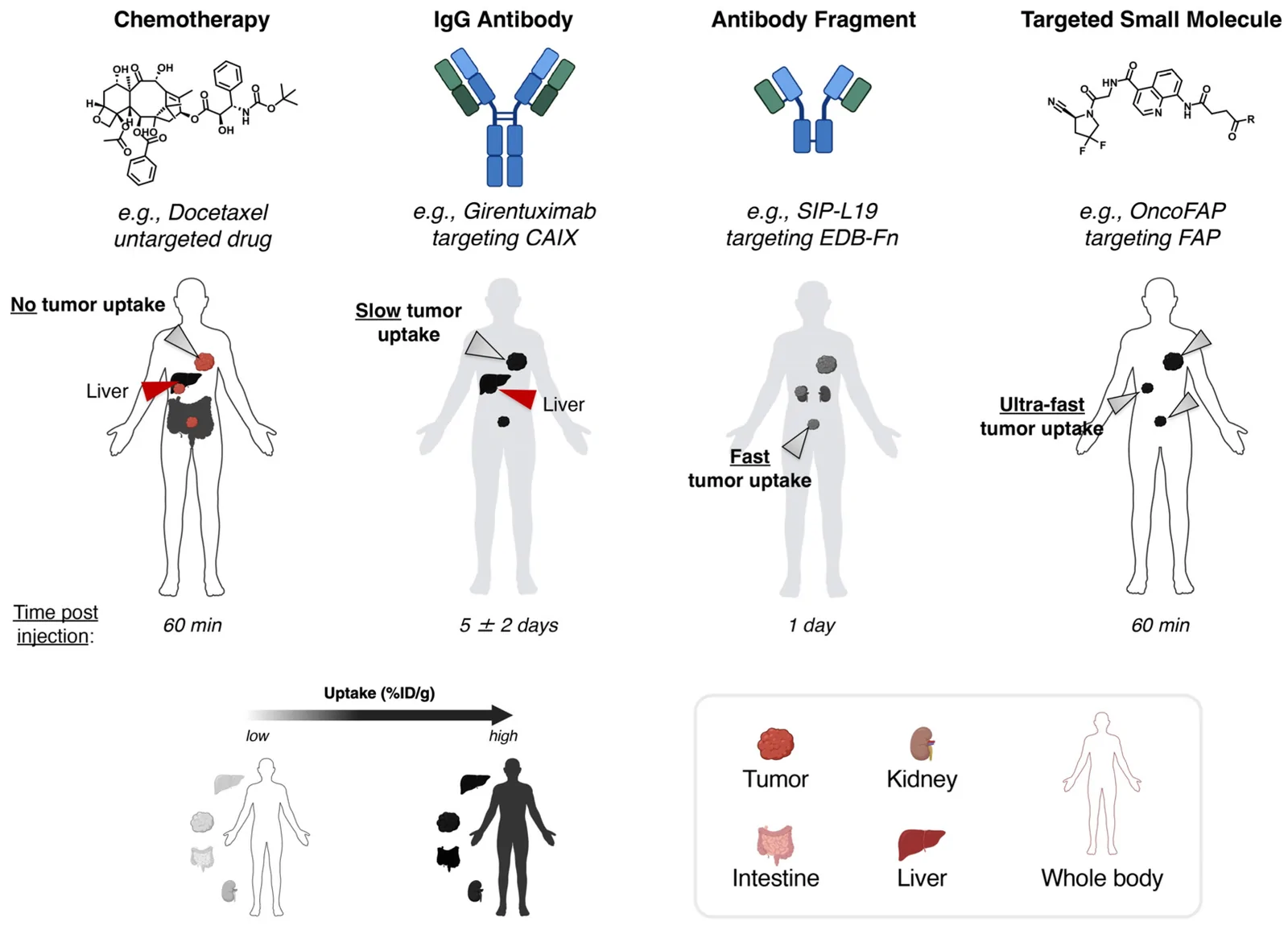
Tumor-targeting antibodies versus small organic ligands. Traditional chemotherapeutic agents, such as Docetaxel, often lack tumor specificity and distribute broadly throughout the body after systemic delivery [2]. As illustrated above for radiolabeled Docetaxel, these agents show minimal preferential uptake in tumor tissue. Tumor-targeting approaches using large biomolecules like monoclonal antibodies (e.g., Girentuximab, which binds CAIX in kidney cancer lesions) or antibody fragments (e.g., Radretumab, which targets EDB-fibronectin) [39,40] offer some degree of specificity but typically suffer from slow tumor penetration and suboptimal pharmacokinetics. In contrast, small-molecule targeting agents can rapidly and selectively home to tumor sites due to their favorable size and binding properties. For instance, the FAP-targeting molecule OncoFAP efficiently localizes to primary and metastatic breast cancer lesions, achieving high tumor-to-background contrast within minutes of administration, and demonstrating strong potential for targeted therapeutic or diagnostic applications [41]. Created in BioRender. Rotta, G. (2026) https://BioRender.com/18eshk5 (accessed on 24 June 2026).

One of the primary advantages of small organic ligands lies in their favorable pharmacokinetics [4,9,10,24]. Unlike antibodies, which typically exhibit prolonged circulation times and limited tumor penetration due to their large size (~150 kDa), small molecules (typically <1 kDa) can rapidly extravasate from the vasculature and diffuse more efficiently and homogeneously into solid tumors, Figure 2 [10,25,26]. Unlike peptides, which tend to accumulate in healthy kidneys, small molecules are rapidly excreted through renal and hepatobiliary routes [27,28,29,30]. This results in faster tissue penetration, higher tumor-to-blood ratios, and reduced systemic exposure, thereby potentially lowering off-target toxicity [10,25,31]. Moreover, small ligands offer synthetic versatility and chemical tunability. Their structures can be engineered to optimize binding affinity, selectivity, and metabolic stability [32].

Compared to mAbs, small molecules are also less immunogenic [33,34] and easier to manufacture [35]. Antibody production requires complex and costly biologics infrastructure, including mammalian cell expression systems and lengthy purification protocols [36]. In contrast, small molecules can be synthesized through well-established organic chemistry methods, allowing for scalable, reproducible, and cost-effective production.

These attributes collectively position small organic ligands as highly attractive alternatives for tumor-targeted delivery of therapeutic payloads. In the context of SMDCs and SMRCs, ligands identified via DELs can be conjugated to cytotoxic drugs or radionuclides using linkers optimized for stability and controlled release [31,37,38]. The resulting conjugates can selectively accumulate in tumors, thereby maximizing therapeutic efficacy while minimizing damage to healthy tissues and driving a paradigm shift in precision oncology.

## 3. From DNA Barcodes to Bioactive Payloads: Small Molecule–Drug Conjugates and Small Molecule–Radio Conjugates

**Figure 3:**
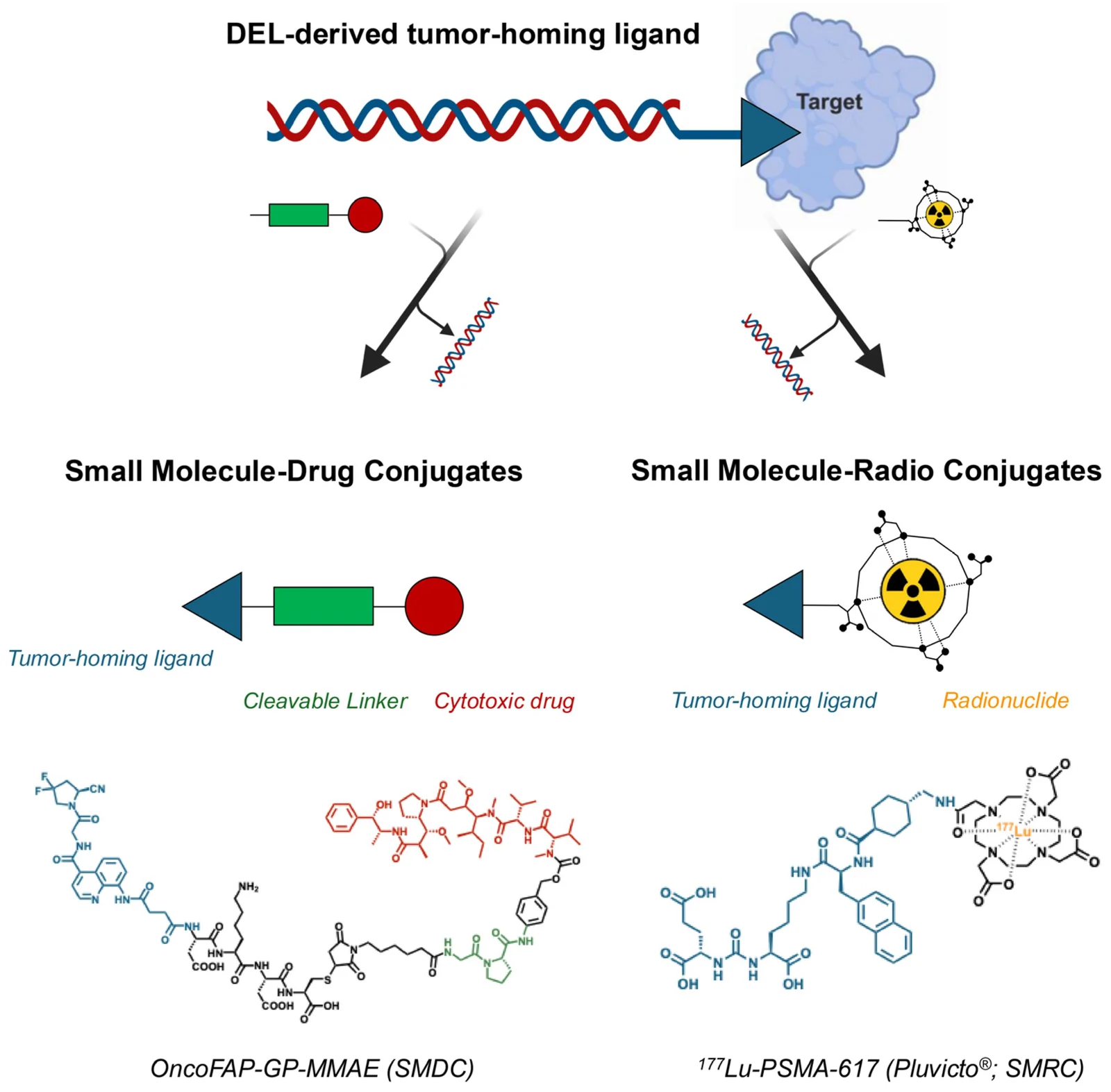
Small molecule–radio and small molecule–drug conjugates. Small molecule therapeutics such as SMRCs and SMDCs can be obtained from DEL-derived ligands (i.e., illustrated as a blue triangle) by replacing the DNA-barcode with a radionuclide payload or with a linker-cytotoxic drug module, respectively. While there are no examples of approved products based on SMDCs [50,51], two SMRC drugs named Lutathera^®^ and Pluvicto^®^ (chemical structure shown in the figure) have been recently approved for the treatment of neuroendocrine tumors and metastatic castration-resistant prostate cancer [53,54]. The structure of OncoFAP-GlyPro-MMAE, a pan-tumoral clinical-stage SMDC prototype, is illustrated in the figure [31]. Tumor-homing ligands are represented in blue (chemical structures and triangles), cleavable linkers are illustrated in green (chemical structures and rectangles), cytotoxic drugs are colored in red (chemical structures and circles), and radionuclides are in orange (chemical structures and radioactive symbol). Created in BioRender. Rotta, G. (2026) https://BioRender.com/fvs61ba (accessed on 24 June 2026).

The discovery of high-affinity ligands through DNA-encoded libraries (DELs) represents only the first step in the transformation from screening hit to therapeutic agent. Once a tumor-selective small molecule is identified and validated, it can be chemically modified to carry a cytotoxic or radioactive payload, thereby forming a small molecule–drug conjugate (SMDC) or small molecule–radio conjugate (SMRC) [4,22,23,25]. These targeted therapeutics aim to deliver potent agents directly to malignant tissues, sparing healthy cells and minimizing systemic toxicity, principles that mirror those behind antibody–drug conjugates (ADCs), but with unique pharmacological and manufacturing advantages [4,9,11]. The modularity of DEL-derived ligands makes them ideal for radiolabeling, as they can be chemically adapted to include cytotoxic drugs, metal chelators, or prosthetic groups, Figure 3. These groups can be introduced in place of the DNA barcode, without compromising binding affinity [23].

In SMDCs, the targeting ligand is conjugated to a cytotoxic drug through a linker, which plays a crucial role in controlling the release of the drug [24,31,38]. Linkers can be designed to remain stable in circulation but cleave under specific intracellular conditions, such as acidic pH [42,43], redox environment [44], or enzymatic activity [31,45], thus ensuring that the payload is preferentially released at the cancer site. The small size and rapid tumor penetration of these conjugates also enable more homogenous drug distribution within solid tumors, overcoming a known limitation of larger biologic-based therapies [10,24,46,47]. This approach has demonstrated efficacy in preclinical settings, particularly for cancers that overexpress specific cell-surface proteins, such as Carbonic Anhydrase IX (CAIX), Prostate-Specific Membrane Antigen (PSMA), or Folate Receptor-α (FR) [46,47,48,49]. While PSMA and FR targeting SMDCs have failed to reach the market due to a lack of efficacy in the clinic [50,51], novel candidates based on high-affinity targeting ligands directed against tumor-associated antigens expressed in the tumor microenvironment, such as Fibroblast Activation Protein (FAP), are being developed and may offer improved therapeutic outcomes by enhancing selective tumor localization, reducing off-target toxicity, and enabling more effective payload delivery [25,31,41].

SMRCs operate under similar principles but employ therapeutic radionuclide payloads, such as Lutetium-177 and Actinium-225, or diagnostic radioisotopes, such as Fluorine-18 and Gallium-68. In the case of SMRCs, the linker between the radionuclide and the tumor-homing ligand is typically designed as an uncleavable structure as the payload does not have to be released to exert its diagnostic or therapeutic function [7]. These radiopharmaceuticals can deliver therapeutic radiation doses directly to tumors (in the case of β- or α-emitters) or enable sensitive molecular imaging (via PET or SPECT tracers). In a “Theranostic approach”, the exact same ligand (for example, discovered by screening DNA-encoded chemical libraries) can be conjugated to a radionuclide payload for imaging (e.g., Gallium-68) or for therapy (e.g., Lutetium-177). These applications are particularly compelling, allowing for real-time assessment of tumor targeting and therapeutic response [52]. This approach has proven successful in the clinic for the treatment of metastatic prostate cancer and neuroendocrine tumors, with the approval of Pluvicto^®^ and Lutathera^®^, two radioligand therapeutics targeting PSMA and somatostatin receptor 2 (sstr2) and based on Lutetium-177 as radionuclide payload [53,54]. A vast number of companion diagnostics and imaging SMRCs have also been approved for the detection of cancer lesions in the same indications [55,56]. The market for radiopharmaceuticals is projected to reach approximately 26.5 billion USD by 2031, with a compound annual growth rate of 14.4% [57]. Notably, all radiopharmaceuticals approved to date are based on ligands discovered through conventional medicinal chemistry approaches. The advent of DELs, combined with a focus on previously uncharted tumor targets, is opening new avenues for the development of next-generation SMRCs.

Advances in ligand discovery, linker chemistry, and radiochemistry are rapidly expanding the scope of tumor-targeted small molecule therapeutics. Combined with the versatility of DEL-derived ligands and the integration of companion imaging diagnostics, SMDCs and SMRCs represent a rapidly maturing class of targeted therapeutics poised to complement or even surpass existing antibody-based approaches in certain oncologic indications.

While the use of these payloads is well established, the application of small-molecule tumor-homing moieties is not limited only to drugs and radionuclides. Cytotoxic compounds and radioactive isotopes have the advantage of being small, thus not significantly impacting the tumor-targeting performance and pharmacokinetic profile of DEL-derived small organic ligands [22,23]. However, a formal demonstration of the maximum molecular weight payload that can be effectively delivered at the site of disease by small ligands is missing. Our group has shown, for example, that nanomolar ligands of Carbonic Anhydrase IX can be used to deliver Interleukin-2 (a small immunomodulatory protein of ~15.5 kDa) to cancer lesions in mice, but with suboptimal tumor-targeting performance [58]. DELs can, in principle, also be applied to discover ligands against immunomodulatory receptors to generate novel payloads that are amenable to conjugation with tumor-targeting ligands [59]. While the field of fully synthetic tumor-targeted immunomodulatory conjugates is in its infancy, this approach holds great promise, especially for the treatment of solid tumors for which protein-based bispecific antibodies are limited by slow extravasation and poor targeting performance.

## 4. Opportunities in Nuclear Medicine

The limitations of conventional monoclonal antibodies for the in vivo delivery of radionuclides for imaging or for therapy applications are well understood. Intact antibodies in IgG format exhibit a long circulatory half-life (typically, 1–2 weeks), which is detrimental both for imaging (suboptimal tumor:organ ratios, prolonged imaging windows required for optimal diagnosis, excessive exposure to radiation) and for therapy (insufficient tumor selectivity, high dose to the bone marrow which typically becomes the limiting organ for toxicity) [60]. Antibody fragments (or other small globular proteins capable of specific binding to accessible tumor-associated antigens) can only partially improve the situation compared to intact antibodies. The shorter circulatory half-life in blood (typically 0.5–2 h) reduces the exposure of normal organs to radiation, but also the absolute antibody uptake in the tumor lesion [61]. Furthermore, small antibody fragments and globular proteins often exhibit significant renal accumulation due to glomerular filtration and proximal tubule reabsorption, which can limit their therapeutic application. Nonetheless, the observation of tumor:organ ratios of approximately 10:1 twenty-four hours after intravenous administration has been reported, both in mouse models of cancer and in patients [40,61,62]. The use of ultra-high-affinity binders, with a molecular weight in the 500–3000 Dalton range, opens extremely attractive opportunities for Nuclear Medicine applications. Those agents extravasate almost instantaneously, thus allowing an efficient and deep penetration into the tumor cell mass [4,10]. Surprisingly, while small ligands have a short circulatory half-life (typically 1–10 min), they may exhibit higher percent of injected dose per gram values in tumors compared to antibodies, in a process which is facilitated by the rapid extravasation [28,41,63,64,65,66]. This efficient targeting process is particularly beneficial when targeting accessible tumor-associated antigens with negligibly low expression in normal organs [41,63,65]. For such applications, DEL technology has opened novel tumor targeting applications, as binding affinity is crucially important for selective and persistent accumulation at the site of disease [22,64,67]. However, when tumor antigens with substantial expression in certain normal organs are considered, the comparative evaluation of antibody-based and small molecule-based targeting is less straightforward. This situation is well exemplified by the targeting of carbonic anhydrase IX (CAIX), a marker of clear-cell renal cell carcinoma (ccRCC). CAIX is virtually undetectable in all normal tissues, exception made for stomach and small intestine, in which a strong staining in immunohistochemistry is detectable. As expected, high-affinity small CAIX ligands efficiently localize both to ccRCC lesions and to CAIX-positive structures (i.e., stomach and small intestine) [66]. By contrast, anti-CAIX antibodies reach ccRCC lesions (albeit slowly) without exhibiting a preferential accumulation in the gastrointestinal tract [60]. This feature is explainable if we assume that intact antibodies extravasate in the leaky tumoral vasculature but not in the intact vasculature of healthy organs, therefore making the antigen “invisible” at those locations.

## 5. Conclusions

DEL technology is revolutionizing the way small organic ligands are discovered. The possibility of creating and screening very large libraries, including affinity-maturation libraries, has already led to the isolation of potent ligands with *K_d_* values in the subnanomolar range, which have progressed to clinical trials for ligand-based pharmacodelivery. As new and improved libraries become available, the contribution of DEL technology to the field of ligand-based pharmacodelivery is expected to continue growing.

At this moment in time, not all targets of pharmaceutical interest can be drugged using DEL technology. Some of the “difficult targets” include tumor-associated antigens, with very flat surfaces and devoid of “clefts”, which would naturally facilitate ligand discovery efforts. It is likely that a new generation of DELs, with judiciously chosen scaffolds, may help overcome some of the technical limitations that still restrict those targets to antibody-based pharmaceutical strategies.

## Data Availability

No new data was created or analyzed in this study. Data sharing is not applicable to this article.
